# Interaction of Cu(II)with His-Val-Gly-Asp and of Zn(II) with His-Val-His, Two Peptides at the Active Site of Cu,Zn-Superoxide Dismutase

**DOI:** 10.1155/S1565363303000086

**Published:** 2003

**Authors:** Alexandra Myari, Gerasimos Malandrinos, John Plakatouras, Nick Hadjiliadis, Imre Sóvágó

**Affiliations:** 1 University of Ioannina Department of Chemistry Ioannina 45110 Greece; 2 University of Debrecen Department of Inorganic and Analytical Chemistry Debrecen H-4010 Hungary

## Abstract

His-Val-His and His-Val-Gly-Asp are two naturally occurring peptide sequences, present at the active site of Cu,Zn-superoxide dismutase (Cu,Zn-SOD). We have already studied the interaction of His-Val-His=A (copper binding site) with Cu(II) and of His-Val-Gly-Asp=B (zinc binding site) with Zn(II). As a continuation of this work and for comparison purposes we have also studied the interaction of Zn(II) with His-Val-His and Cu(II) with His-Val-Gly-Asp using both potentiometric and spectroscopic methods (visible, EPR, NMR). The stoichiometry, stability constants and solution structure of the complexes formed have been determined. Histamine type of coordination is observed for/ZnAH/^2+^, /ZnA/^+^, /ZnA^2^H/^+^ and/ZnA^2^/ in acidic pH while deprotonation of coordinated water molecules is observed at higher pH. /CUB/ species is characterized by the formation of a macrochelate and histamine type coordination. Its stability results in the suppression of amide deprotonation which occurs at high pH resulting in the formation of the highly distorted from square planar geometry 4N complex/CuBH_-3_/^3^.


### Interaction of Cu(II) with His-Val-Gly-Asp and of Zn(II) with His-Val-His, Two Peptides at the Active Site of Cu,Zn-Superoxide Dismutase

Alexandra Myari,[a] Gerasimos Malandrinos,[a] John Plakatouras,[a] Nick Hadjiliadis[a,*]

and Imre Sóvágó[b]

[a] *University of Ioannina, Department of Chemistry, Ioannina 45110, Greece*
[b] *University of Debrecen, Department of Inorganic and Analytical Chemistry, H-4010, Debrecen, Hungary*

(Received: August 12, 2002; Accepted: September 18, 2002)

### ABSTRACT

His-Val-His and His-Val-Gly-Asp are two naturally occurring peptide sequences, present at the active site of Cu,Zn-superoxide dismutase (Cu,Zn-SOD). We have already studied the interaction of His-Val-His=A (copper binding site) with Cu(II) and of His-Val-Gly-Asp=B (zinc binding site) with Zn(II). As a continuation of this work and for comparison purposes we have also studied the interaction of Zn(II) with His-Val-His and Cu(II) with His-Val-Gly-Asp using both potentiometric and spectroscopic methods (visible, EPR, NMR). The stoichiometry, stability constants and solution structure of the complexes formed have been determined. Histamine type of coordination is observed for $/ZnAH/^{2+}$, $/ZnA/^{+}$, $/ZnA_2H/^{+}$ and $/ZnA_2/$ in acidic pH while deprotonation of coordinated water molecules is observed at higher pH. /CuB/ species is characterized by the formation of a macrochelate and histamine type coordination. Its stability results in the suppression of amide deprotonation which occurs at high pH resulting in the formation of the highly distorted from square planar geometry 4N complex $/CuBH_{-3}/^{3-}$.

### INTRODUCTION

Oxidative stress is implicated in numerous biological processes such as amyotrophic lateral sclerosis, rheumatoid arthritis, aging, carcinogenesis and inflammation /1,2/. Superoxide dismutases comprise an integral part of the cellular protective mechanism against oxidative stress. Cu,Zn superoxide dismutase

*To whom correspondence should be addressed.
Phone: xx30·6510·98420, Fax: xx30·6510·44831
e-mail: nhadjil@cc.uoi.gr

(Cu,Zn-SOD), found in all eukaryotic cells, catalyses the disproportionation of superoxide anion radical through the two step reaction /3/:

$$O_2^- + Cu^{II}Zn^{II}SOD \xrightarrow{k_1} O_2 + Cu^{I}Zn^{II}SOD \quad (1)$$

$$O_2^- + 2H^+ + Cu^{I}Zn^{II}SOD \xrightarrow{k_2} H_2O_2 + Cu^{II}Zn^{II}SOD \quad (2)$$

The active center of Cu,Zn-SOD, as shown by crystallographic data /4/, consists of one copper ion and one zinc ion, bridged by the imidazolate ring of a histidine residue of the protein backbone. Copper is coordinated to four histidine residues ($His_{44}$, $His_{46}$, $His_{61}$, $His_{118}$) and a water molecule in a distorted square planar geometry whereas zinc is coordinated to three histidine ($His_{61}$, $His_{69}$, $His_{78}$) and one aspartate residue ($D_{81}$) in a distorted tetrahedral geometry. Copper is very important in maintaining superoxide dismutase activity whereas the replacement of zinc with other metals like $Cu^{2+}$, $Co^{2+}$, $Hg^{2+}$, $Cd^{2+}$, $Ni^{2+}$ or $VO^{2+}$ does not affect the enzymatic activity. The role of zinc in the function of SOD is not apparent and it is suggested that it plays a structural role. On the other hand, the fact that point mutations at the zinc site account for approximately 2% of cases of amyotrophical lateral sclerosis is suggestive of a modulating role for zinc /5/.

The design and the application of synthetic low molecular weight metal complexes as SOD mimics have received considerable attention during the last decades. Effectiveness, permeability of cell membranes, low cost and low toxicity are considered to be essential properties for an efficient SOD mimic. A great number of mononuclear, homo- and heterodinuclear metal complexes with ligands such as Schiff bases /6/, macrocyclic ligands /7/ and peptides /8/ have been proposed as SOD mimics.

Our focus has been the incorporation of peptides, part of the native sequence, in the design of a structural analogue of the Cu,Zn-SOD. $His_{44}$-$Val_{45}$-$His_{46}$ is part of the copper active center participating in coordination through the imidazole groups of the two histidine residues. $His_{78}$-$Val_{79}$-$Gly_{80}$-$Asp_{81}$ is present in the zinc active site coordinating to the zinc ion through the imidazole group of the histidine residue and the carboxylate group of the aspartate residue. In previous work /9/, we have characterized the species formed in the systems His-Val-His-Cu(II) and His-Val-Gly-Asp-Zn(II). As a continuation of this work, we have also studied the interaction of His-Val-His with Zn(II) and His-Val-Gly-Asp with Cu(II) and compared them with the previous ones.

### EXPERIMENTAL

### Materials

All solvents and chemicals from commercial sources, used for synthesis, were of the highest available purity and used without further purification. The protected amino acids Fmoc-His($N^{im}$-Mtt)-OH, Fmoc-Val-OH, Fmoc-Gly-OH and Fmoc-Asp(O-$Bu^t$) (Fmoc = Fluoren-9-ylmethoxycarbonyl, Mtt = 4-methyltrityl, O-$Bu^t$ = tertiary-Butyl) and the resin 2-chlorotrityl chloride were from CBL Chemicals Ltd., Patras, Greece.

$CuCl_2$ and $Zn(NO_3)_2$ were of analytical grade from Merck. Metal ion stock solutions were prepared and used and the concentrations were checked gravimetrically *via* oxinates.

### Peptide synthesis

The peptides His-Val-His and His-Val-Gly-Asp were synthesized as previously described /9/.

### Methods

(i) The pH-metric titrations in the pH range 2.0-10 were performed in 5 $cm^3$ samples in the concentration range of 2-4x$10^{-3}$ mol $dm^{-3}$ at ligand to metal ion ratios 1:1 and 2:1. The number of experimental points was 100-120 ($cm^3$-pH) for each titration curve. In the case of the system His-Val-His-$Zn^{2+}$ 1:1, experimental data points for pH>6.0 had to be rejected because of precipitation of metal hydroxide. Argon was bubbled through the samples to ensure the absence of oxygen and carbon dioxide and for stirring the solutions. All pH-metric measurements were carried out at 298 °K, at a constant ionic strength of 0.2 mol $dm^{-3}$ KCl. The potentiometric titrations were carried out using a Radiometer PHM 84 pH-meter, equipped with a 6.0234.100 combined electrode (Metrohm) and a Dosimat 715 burette (Metrohm). The pH-readings were converted to hydrogen ion concentration as described elsewhere /10/ and the value $pK_w$=13.75 was used for the ionization of water. The evaluation of the measurements and calculations of the stability and ionization constants were performed, using the pHENG /11/, PSEQUAD /12/ and SUPERQUAD /12/ computer programs.

(ii) The pH values of the samples for spectroscopic studies were measured with a Grison GLP21 pHmeter, equipped with a glass electrode || Ag/AgCl, U402-M6-57/100 of Mettler, Toledo.

(iii) The visible spectra were recorded on a Jasco 530 spectrophotometer, in the same concentration range as used for potentiometry.

(iv) The EPR continuous wave spectra were recorded at liquid He temperature in a EP-200D Bruker instrument at the X-band, equipped with a cryostat of Oxford Instruments.

(v) The $^1H$ NMR spectra were recorded on a Bruker AMX 400 MHz spectrometer at 300 °K. Deuterium oxide was used as a solvent. Concentration and ionic strength were of the same range as used for the potentiometric measurements.

### RESULTS AND DISCUSSION

### A. Characterization of the system His-Val-His (A)-Zn(II).

Acid dissociation constants of His-Val-His have been previously determined /9/. Formation constants for the zinc(II)-His-Val-His complexes were evaluated from titration data in the pH range 4-12. The stability constants for the species formed are given in Table 1. For comparison purposes the formation constants for Cu(II) complexes with His-Val-His /9/ are also shown. Cu(II) complexes with His-Val-His are thermodynamically more stable than the Zn(II) complex as expected, according to the Irving-Williams series.

Ligand excess is required to keep zinc in solution in alkaline media as zinc hydroxide precipitates at pH>6 in the 1:1 system. For the system of His-Val-His-Zn(II) 2:1 it is found that His-Val-His forms numerous complexes with zinc(II) ion, all of them present over a wide pH range, making it hard to evaluate each species separately. Species distribution for the system His-Val-His-Zn(II) 2:1 is shown in Figure 1.

**Table 1**

Formation Constants of $Zn^{II}$ and $Cu^{II}$-His-Val-His complexes[α,β].

| | $\log\beta_{112}$ | $\log\beta_{111}$ | $\log\beta_{110}$ | $\log\beta_{122}$ | $\log\beta_{121}$ | $\mathrm{Log}\beta_{120}$ | $\log\beta_{12\text{-}1}$ | $\log\beta_{11\text{-}1}$ | $\log\beta_{11\text{-}2}$ |
|---|---|---|---|---|---|---|---|---|---|
| Zn(II) | - | 11.65 (2) | 5.94 (3) | - | 16.65 (7) | 9.85 (3) | 1.48 (3) | - | -12.12 (4) |
| Cu(II) | 18.56 | 15.41 | 10.53 | 28.08 | 21.53 | - | - | 4.33 | -2.73 |

[α] Formation constants are defined by eq. 1 and eq.2 where L is the fully deprotonated ligand:

$pZn + qL + rH \rightleftarrows Zn_pL_qH_r$ (1), $\beta_{pqr} = [Zn_pL_qH_r]/[Zn]^p[L]^q[H]^r$ (2).

[β] Standard deviation is indicated in the parenthesis.

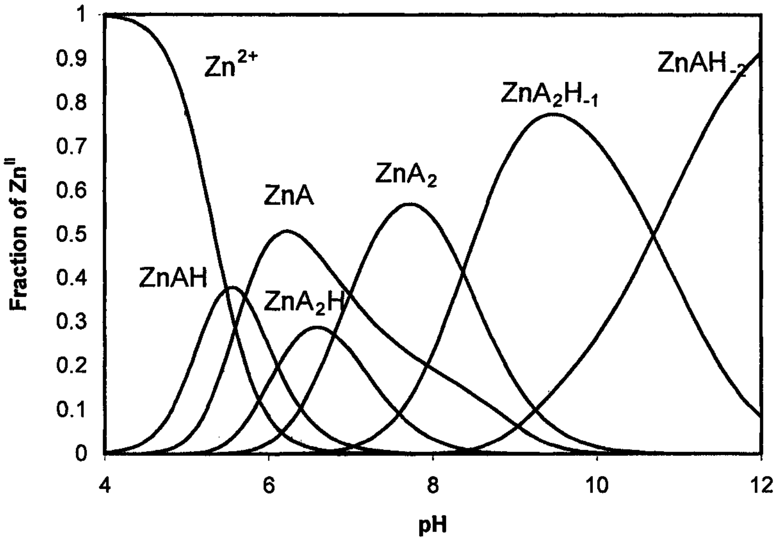

**Fig. 1:** Species distribution for the system His-Val-His-Zn(II) 5mM:2.5 mM (2:1).

The presence of histidine in the N-terminal position favors histamine type coordination forming a $\{NH_2, N_{im}\}$ chelate [13]. Coordination of the terminal amino group and the N-terminal imidazole is possible for the ZnAH species, with the C-terminal imidazole remaining protonated. The pK value for the deprotonation and coordination of the C-terminal imidazole (pK=5.71) for His-Val-His is close to the one observed for His-Pro-His (pK=6.12) [14]. Furthermore, the ZnA species exhibits a slightly increased stability ($\log\beta_{110}$=5.43) compared to the simple model of histamine ($\log\beta_{110}$=5.15) [15] suggesting the formation of a macrochelate *via* the coordination of the C-termirıl imidazole.

The formation constant for the species $ZnA_2$ ($\log\beta_{120}$=9.85) corresponds well to the one observed for histamine ($\log\beta_{120}$=9.97) [15] suggesting histamine type of coordination for both peptide molecules. The pK value for the ionization of $ZnA_2H$ to $ZnA_2$ (pK=6.80) approximates the pK value of the C-terminal imidazole of the free peptide (pK=6.73) suggesting that the C-terminal imidazole of the one peptide molecule in the $ZnA_2H$ species remains protonated.

Upon titration of the system HVH-$Zn^{II}$ 2:1, two additional equivalents of protons were titrated per metal ion. This is in agreement with what was observed before for the titration of similar systems /9,16/. The possibilities for the groups from which the additional equivalents of protons are titrated include coordinated water molecules and the peptide imino groups. The pK value ($ZnA_2 \rightarrow ZnA_2H_{-1} + H^+$ pK=11.33) is of the same magnitude as for the titration of a proton from a coordinated water molecule in the Zn(II) complexes of nitrilotriacetic acid and β, β', β''-triaminotriethylamine (10.0 and 11.1, respectively) /17/. Further deprotonation of the $ZnA_2H_{-1}$ complex may lead to the displacement of one of the two ligands by a second hydroxide ion, resulting in complex $ZnAH_{-2}$.

To elucidate further the nature of complexes of Zn(II) with His-Val-His, $^1H$ NMR spectra of His-Val-His and His-Val-His-Zn(II) 2:1 in $D_2O$ were measured. Figure 2 shows the imidazole region for the system His-Val-His-Zn(II) 2:1 as a function of pD. Comparison of the chemical shifts for the free peptide and in the presence of Zn(II) at selected pD values is further illustrated in Figure 3.

From Figure 2, it can be concluded that the complexation of His-Val-His by Zn(II) is pD-dependent. In acidic pD, the resonances for the imidazole protons are shifted downfield upon complexation of the peptide

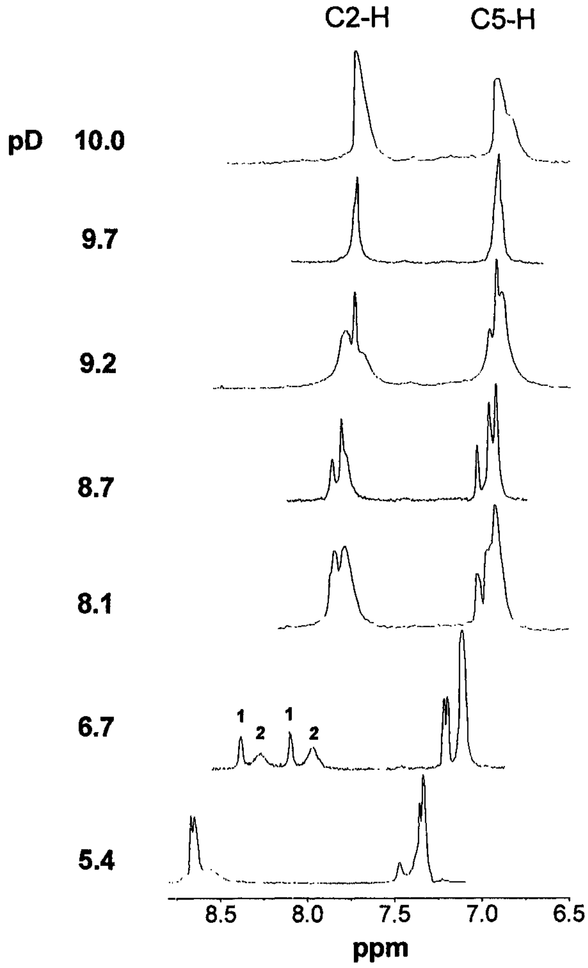

**Fig. 2:** Imidazole region of $^1H$ NMR spectra of $D_2O$ solutions containing 10 mM His-Val-His, 5 mM $Zn(NO_3)_2$ and 0.2 M KCl, as a function of pD.

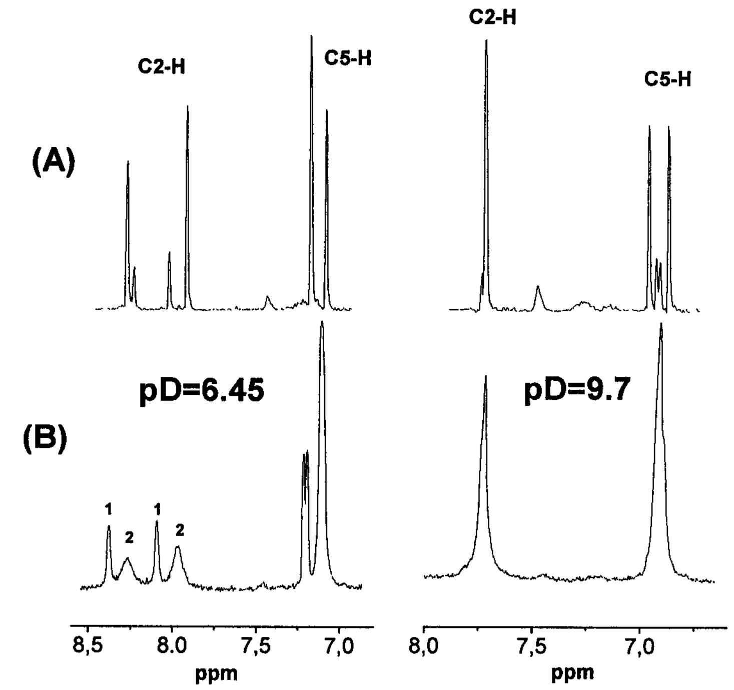

**Fig. 3:** Comparison of the imidazole region of $^1H$ NMR spectra of free His-Val-His (A) and the system His-Val-His-Zn(II) 2:1 at several pD values.

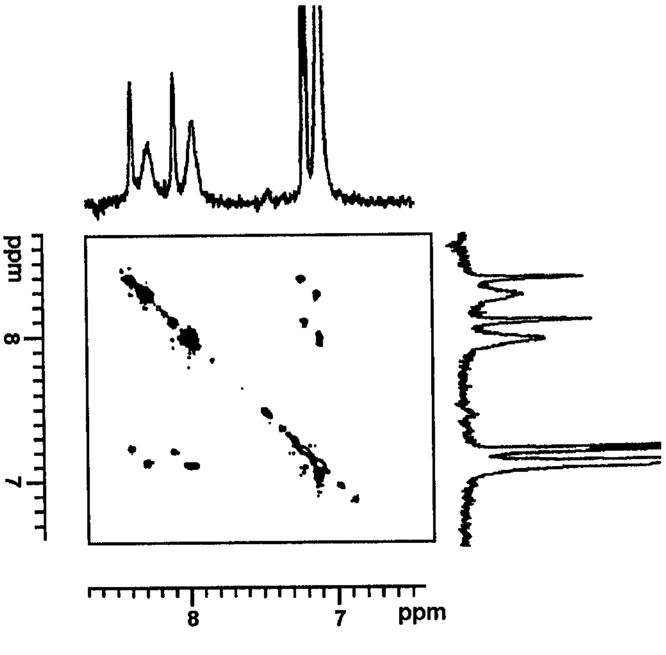

**Fig. 4:** $^1H$ NMR TOCSY imidazole region of the $D_2O$ solution of His-Val-His-Zn(II) 2:1 at pD=6.5.

with Zn(II). A second set of resonances is detectable at pD=5.4 which increases as pD increases. At pD=6.7 the two sets of resonances (**1** and **2**) are well-resolved confirming the presence of two different species. This is further supported by the two dimensional spectrum $^1H$-$^1H$ TOCSY (Figure 4) in which each set of resonances of the C2-H imidazole protons corresponds to a set of resonances of C5-H imidazole protons.

The first species (1) appears to be kinetically stable on the NMR time scale whereas the second one (2) exhibits relative broad resonances possibly due to fast exchange of the peptide between the free and complexed form. The presence of two species exhibiting different kinetic behavior had been observed previously for complexes of Zn(II) with the peptides Gly-His, Gly-Ala [18], Gly-His-Gly and Gly-His-Lys [19]. In these studies, the binding of Zn(II) in the kinetically stable complex was found to occur *via* the N-terminal amino nitrogen, the deprotonated amide nitrogen, and the imidazole 1-nitrogen. The differences in the chemical shifts of Table 2 demonstrate that this is not the case for the Zn(II)-His-Val-His system. There is a difference in the chemical shifts of C2-H protons upon complexation of the imidazole and His α-CH protons upon binding of the terminal amino group at pD=6.5. In the case of Zn-Gly-His and Zn-Ala-His systems, the shift of 0.156 ppm and 0.212 ppm to lower frequency respectively for the α-CH protons was not observed for the system Zn-His-Val-His.

The species distribution of the Zn-His-Val-Gly-Asp 1:2 system can give us an insight in the interpretation of the NMR data. In ZnA and ZnAH, present in solution at pD=6.5 (Figure 1), at least two nitrogen atoms coordinate to zinc ion, whereas in the species $ZnA_2H$ and $ZnA_2$, also present at pD=6.5, the mode of coordination is {4N}. This suggests the presence of two different types of species with different coordination modes at this pH. Comparison of the integral of the peaks for complex **1** (46%) and **2** (54%) with the fractions of MA, MAH (48%) and $MA_2$, $MA_2H$ (51%) calculated by potentiometric data shows an agreement within 5% deviation.

**Table 2**

$^1H$ NMR chemical shifts of His-Val-His and zinc complexes of His-Val-His.

| pD | System | C2-H | C4-H | His α-CH | Val α-CH |
|---|---|---|---|---|---|
| 6.5 | His-Val-His | 8.517, 8.081 | 7.212, 7.178 | 4.540, 4.298 | 4.196 |
| 6.5 | $Zn^{II}$-HVH | 8.387, 8.270 8.098, 7.968 | 7.219, 7.199, 7.114 | 4.455, 4.340 | 4.165 |
| 9.7 | His-Val-His | 7.741, 7.724 | 6.983, 6.877 | 4.467, 3.815 | 4.140 |
| 9.7 | $Zn^{II}$-HVH | 7.739 | 6.929 | 4.429, 3.796 | 4.115 |

**Table 3**

Formation Constants of His-Val-Gly-Asp-$Cu^{II}$ and $Zn^{II}$ complexes$^{\alpha,\beta}$.

| | $\log\beta_{112}$ | $\log\beta_{111}$ | $\log\beta_{110}$ | $\log\beta_{121}$ | $\log\beta_{120}$ | $\log\beta_{11-1}$ | $\log\beta_{11-2}$ | $\log\beta_{11-3}$ |
|---|---|---|---|---|---|---|---|---|
| Cu(II) | - | 13.85 (1) | 9.74 (1) | 20.86 (7) | 15.08 (3) | 1.89 (2) | -7.21 (2) | -17.60 (2) |
| Zn(II) | 15.55 | 10.90 | 5.43 | 16.83 | 10.05 | -2.76 | -12.34 | -23.18 |

$^{\alpha}$ Formation constants are defined by eq. 1 and eq.2 where B is the fully deprotonated ligand:

$pCu + qB + rH \rightleftarrows Cu_pB_qH_r$ (1), $\beta_{pqr} = [Cu_pB_qH_r]/[Cu]^p[B]^q[H]^r$ (2).

$^{\beta}$ Standard deviation x$10^{-2}$ is indicated in the parenthesis.

As is illustrated in Figure 2, resonances of the imidazole protons are pD independent above pD=8.1 whereas the chemical shifts for imidazole and His α-CH protons (Table 2) approximate the chemical shifts of the uncomplexed peptide. This is due to the presence of hydroxide ions partially displacing the peptide donor atoms /17/. The proposed structures for the species formed are shown in Figure 5.

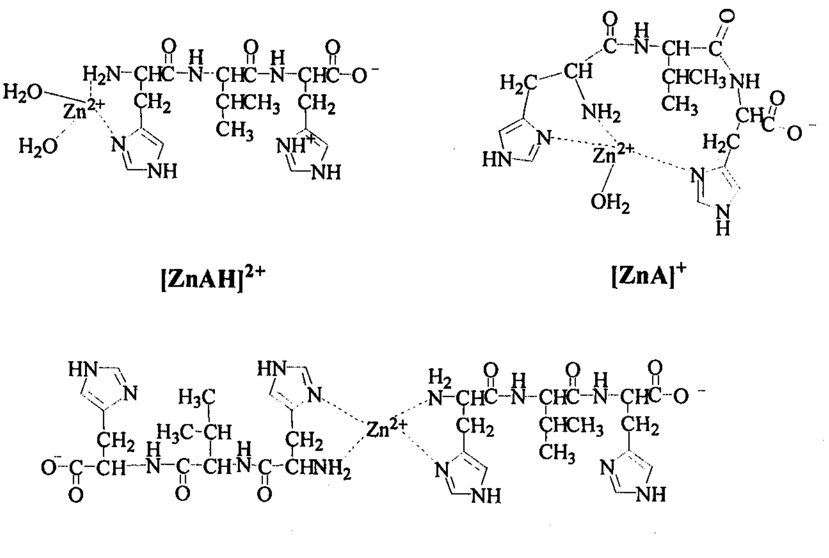

$[ZnAH]^{2+}$ $[ZnA]^{+}$

$[ZnA_2]$

**Fig. 5:** Proposed structures for the species formed in the system Zn(II)-His-Val-His.

### B. Characterization of the system His-Val-Gly-Asp(B)-Cu(II).

Acid dissociation constants have been determined previously [9]. The species formed were characterized by potentiometric titrations in the pH range 3-11, EPR spectroscopy and visible spectra. The formation constants of the complexes are shown in Table 3. Stability constants for Zn(II) complexes [9] are also shown for comparison purposes.

The species distribution for the systems 1:1 and 2:1 are shown in Figure 6. The presence of histidine at the N-terminus results in histamine type coordination also for this peptide [13]. CuBH is a dominating species at pH~3.5 (Figure 6) probably having the C-terminal carboxylate, the aspartyl sidechain carboxylate and the N-terminal histidine imidazole deprotonated. The N-terminal amino group remains protonated at this low pH range excluding the possibility of histamine type of coordination. This is further supported by visible spectroscopy since CuBH exhibits a $\lambda_{max}$=744 nm (Table 4) which is much higher compared to the Cu(II)-histamine system ($\lambda_{max}$=681 nm) [20] suggesting that coordination in CuBH takes place *via* one nitrogen donor atom, probably from the N-terminal imidazole. However, an equilibrium between species with different coordination modes, comprising the species with (i) {$NH_2$, CO} coordination at the N-termini with protonated imidazole, (ii) monodentate imidazole coordination with protonated amino group and (iii) histamine-like coordination with protonated beta-carboxylic group, cannot be excluded.

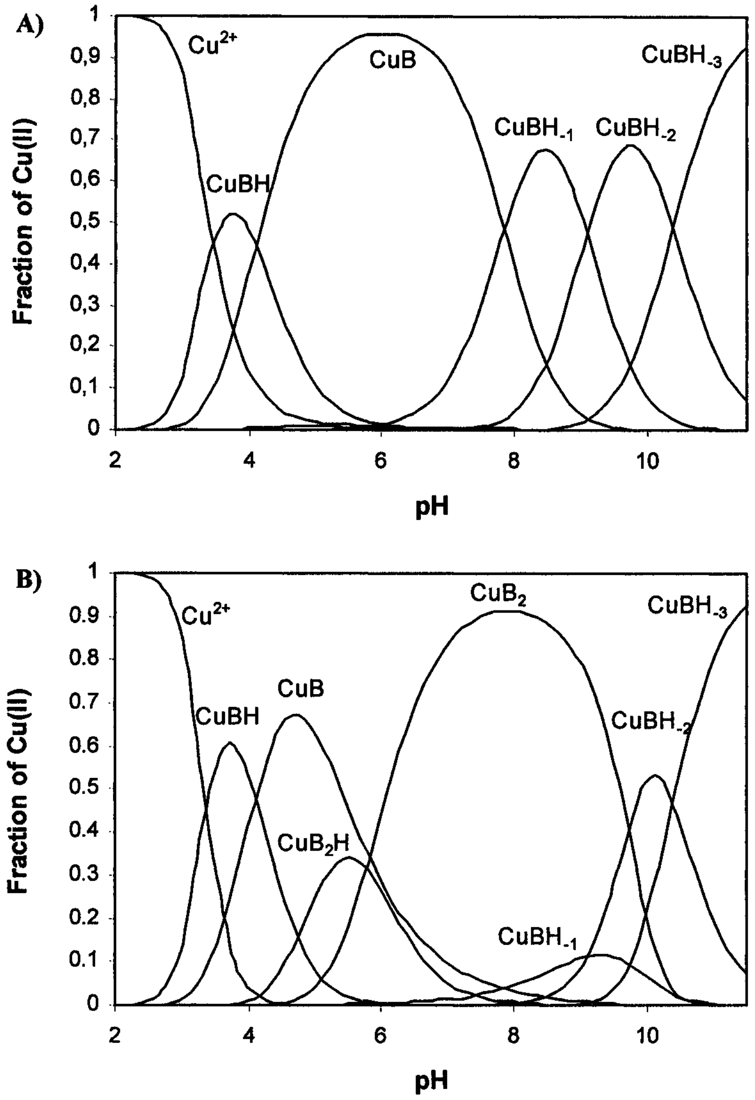

**Fig. 6:** Species distribution for the system His-Val-Gly-Asp(B)-Cu(II) 1:1 **(A)** and 2:1 **(B)**.

In the 1:1 system, a species of 1:1 stoichiometry is present over a wide pH range, 3-9. This species was found to be EPR silent. Potentiometry cannot distinguish whether monomeric or dimeric species is formed since the introduction of the dimeric species into the model did not result in any change of the log β values for the other species formed. The formation of dimeric species of Cu(II) with peptides having histidine at the N-termini like His-His /24/, His-Gly and His-Gly-Gly /20, 22, 23/ has been observed previously. In these dimeric species, the amino group, the deprotonated amide and the carboxylate (His-Gly, His-Gly-Gly) or imidazole (His-His) participate in coordination, whereas the imidazole acts as the bridging group. This mode of coordination is not supported by the present spectroscopic data. The $\lambda_{max}$=672 nm found for the system of His-Val-Gly-Asp approximates the corresponding value found for the system Cu(II)-His-Gly ($\lambda_{max}$=677 nm) /20/, suggesting a histamine type coordination for the 1:1 stoichiometry species. Furthermore, the higher ratio of the stepwise stability contants ($\log K_{CuB}/K_{CuB2}$=4.4) compared to the simple system of histamine ($\log K_{CuB}/K_{CuB2}$=2.98) /15/ suggests extra stabilization of the complex probably coming from the aspartyl side

**Table 4**

Spectroscopic data and proposed coordination modes for the species formed in the system His-Val-Gly-Asp-Cu(II).

| Species[α] | $\lambda_{max}$ (nm) | E ($l \cdot mol^{-1} \cdot cm^{-1}$) | $A_{\|\|}$ ($10^{-4}$ $cm^{-1}$) | $g_{\|\|}$ | Possible donor atoms |
|---|---|---|---|---|---|
| CuBH | 744 | 25 | β | β | - |
| CuB | 672 | 52 | β | β | {$NH_2$, $N_{im}$, $COO^-$} |
| $CuB_2$ | 638 | 97 | 180 | 2.29 | {$2NH_2$, $2N_{im}$} |
| $CuBH_{-1}$ | 608 | 68 | 190 | 2.22 | {$NH_2$, $N_{im}$, $N^-$} |
| $CuBH_{-2}$ | 556 | 99 | β | β | {$NH_2$, $2N^-$, $COO^-$} |
| $CuBH_{-3}$ | 524 | 150 | 175 | 2.26 | {$NH_2$, $3N^-$} |

[α] The measurements were conducted at the pH of equilibrium maximum for each species.

[β] EPR parameters not determined due to poor EPR resolution.

chain carboxylate. This evidence does not support formation of a dimer because in this case the carboxylate acts as a bridging ligand and a 7-membered chelate is formed, not stable enough to provide the extra stabilization required for the dimeric species. Furthermore, potentiometric and spectroscopic studies of the interaction between Cu(II) and peptides having aspartic acid residues have not detected the presence of such dimeric species [24]. The distortion in the geometry of a macrochelate could account for the poor resolution of the EPR spectrum.

The stability of CuB species excludes the formation of the bis complex in the 1:1 system but it cannot be prevented in the case of the 2:1 system in which it is a prevailing species at the pH range 6-9.5. The formation constant for the $CuB_2$ species ($\log\beta_{120}$=15.08) is in good agreement with the one found for Cu(II)-His-Gly system ($\log\beta_{120}$=15.06) [23], for which histamine type of coordination for both peptide molecules has been proposed. On the other hand, the d-d transition for this species centered at 638 nm seems relatively high for a $2NH_2$, $2N_{im}$ coordination. The EPR parameters determined for this species ($g_{||}$ =2.29, $A_{||}$=180) however are in good agreement with similar species formed by ligands containing histamine-like donor sets [15].

In the 2:1 system, $CuB_2H$ is also present at the pH range 4-8. Histamine-type of coordination is made possible for the one peptide molecule whereas the other one acts as a monodentate ligand, probably *via* the imidazole nitrogen atom.

Amide deprotonation is suppressed by the formation of the macrochelate and the bis complexes in the 1:1 and 2:1 system respectively, shifted to higher pH values, having $pK_1$=7.85 for the first amide ionization compared to $pK'_1$=5.56 for the simple system of tetraglycine [25]. A blue shift is noticeable in the visible spectra as the pH increases (Table 4) suggesting the successive deprotonation and coordination of the amide nitrogens. The $\lambda_{max}$=608 nm observed for $CuBH_{-1}$ is similar to that found for His-Gly and His-Gly-Gly ($\lambda_{max}$=613 nm) [22]. This absorption maximum is attributed to the coordination of three nitrogen atoms: the deprotonated amide nitrogen, the terminal amino group and the imidazole. The coordination of the aspartyl side chain carboxylate by formation of a macrochelate cannot be excluded. This evidence is further supported by the EPR parameters ($g_{||}$ =2.22, $A_{||}$=190) which correspond well to a 3N species [25]. However, the three nitrogen donors cannot coordinate equatorially to Cu(II) due to steric hindrance which probably results in the axial coordination of one nitrogen.

The deprotonation and coordination of the second amide nitrogen results in the formation of $CuBH_{-2}$. Spectroscopic data for $CuBH_{-2}$ ($\lambda_{max}$=556 nm) agrees well with the same species formed by triglycine ($\lambda_{max}$=553 nm) having {$NH_2$, $N^-$, $N^-$, $COO^-$} coordination mode /25/. $CuBH_{-2}$ in the system of His-Val-Gly-Asp ($\log\beta_{11-2}$ =-7.21) exhibits extra stabilization compared to the system of tetraglycine ($\log\beta_{11-2}$= -7.41) in which the terminal carboxylate does not participate in the coordination of Cu(II) whereas in the system of triglycine, $CuBH_{-2}$ ($\log\beta_{11-2}$=-7.02) appears to be more stable due to the coordination of the C-terminal carboxylate which is in closer proximity compared to His-Val-Gly-Asp /25/.

The d-d transition for $CuBH_{-3}$ ($\lambda_{max}$=524 nm, $\varepsilon$=150 $l\cdot mol^{-1}\cdot cm^{-1}$ ) is typical for a 4N species formed *via* the coordination of three amide nitrogens and the amino group, in good agreement with the corresponding species of Ala-Ala-Ala-Asp ($\lambda_{max}$=520 nm, $\varepsilon$=140 $l\cdot mol^{-1}\cdot cm^{-1}$) /24/. The EPR parameters ($g_{||}$=2.17, $A_{||}$=175) correspond to 4N coordination mode as found already for other similar systems like Ala-Ala-Ala-Asp ($g_{||}$=2.18, $A_{||}$=205) /24/ and tetraglycine ($g_{||}$=2.172, $A_{||}$=213) /25/ for {$3N^-$, $NH_2$} mode of coordination. The relatively low hyperfine constant compared to what it is found for the square planar 4N donor complexes of Cu(II) with cyclam ($g_{||}$=2.187, $A_{||}$=205) and dioxycyclam ($g_{||}$=2.171, $A_{||}$=215) /25/, reveals a great distortion from the square planar geometry for the $CuBH_{-3}$ complex. On the other hand, the axial interaction of the imidazole may account for this unusually low $A_{||}$ value.

The proposed structures for the complexes formed in the Cu(II)-His-Val-Gly-Asp system are shown in Figure 7.

From the study of the interaction of Cu(II) with the peptide sequences His-Val-His /9/ and His-Val-Gly-Asp, present in the active site of Cu,Zn-SOD, it can be concluded that Cu(II) forms stable complexes with both peptides. In CuL species (L=His-Val-His or His-Val-Gly-Asp), the functional groups present in the native enzyme participate also in the coordination of Cu(II) in solution by both peptides. The comparison of the stability of these two species reveals a higher stability of almost one order of magnitude for His-Val-His ($\log\beta$=10.53) compared to His-Val-Gly-Asp ($\log\beta$=9.74), probably responsible for keeping Cu(II) in its naturally occurring position. The 4-5 orders of magnitude greater stability of Cu(II) complexes with both peptides compared to Zn(II) (Tables 1 and 3) explains well why Cu(II) can replace successfully Zn(II) in the Cu,Zn-SOD enzyme.

On the other hand, ZnL species is approximately 0.5 units more stable for His-Val-His compared to His-Val-Gly-Asp (Tables 1 and 3) probably due to the higher stabilizing effect of the C-terminal imidazole. It is apparent that there is no higher preference for the presence of Zn(II) at the zinc site. This further supports a modulating role of Cu(II) in the activity of the superoxide dismutase enzyme whereas Zn(II) is restricted to a structural role.

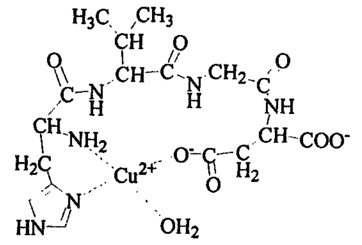

**[CuB]**

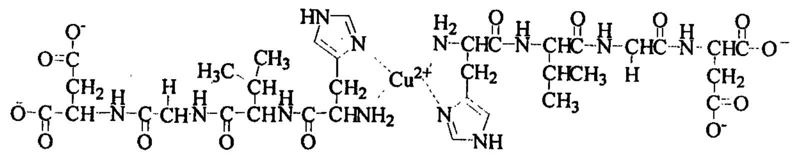

**$[CuB_2]^{2-}$**

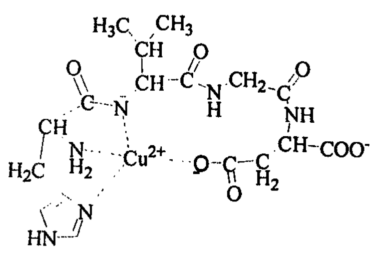

**$[CuBH_{-1}]^-$**

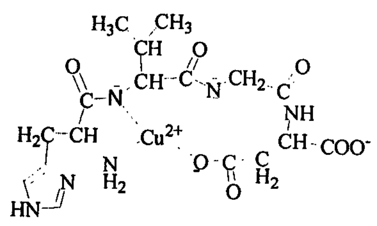

**$[CuBH_{-2}]^{2-}$**

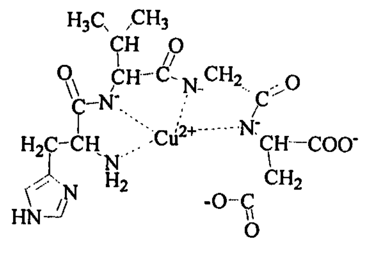

**$[CuBH_{-3}]^{3-}$**

**Fig. 7:** Proposed structures for the species formed in the Cu(II)-His-Val-Gly-Asp system.

### CONCLUSION

We have studied the interaction of Cu(II) with the tetrapeptide His-Val-Gly-Asp and of Zn(II) with the tripeptide His-Val-His. The two peptides surround Cu(II) and Zn(II) ions in the active site of Cu,Zn-SOD. The equilibrium of species in solution was established by potentiometric titrations while the structures of these species were characterized by UV-vis, EPR and $^1$H NMR scpectroscopic measurements. The major binding sites involve the {$NH_2$, $N_{im}$} of the N-terminal histidine for both His-Val-His and His-Val-Gly-Asp. This binding mode was identified in ZnAH, ZnA, $ZnA_2H$, $ZnA_2$ and CuB, $CuB_2$ and $CuBH_{-1}$. At low pH the formation of a kinetically stable and a kinetically labile Zn(II) complexes was demonstrated by NMR whereas at high pH deprotonation of water molecules coordinated to Zn(II) occurs. The stabilizing effect of the aspartyl side chain of His-Val-Gly-Asp results in the formation of a macrochelate in the species CuB. The deprotonation and coordination of amide nitrogens cannot be prevented and occurs at high pH leading to the formation of complexes with great distortion of square planar geometry. Our next step concerns the evaluation of the importance in SOD activity of these different coordination modes observed.

### REFERENCES

1. B. Halliwell and J. M. C. Gutteridge, *Biochem. J,.* **219**, 1 (1984).
2. J. G. Scandalis (Ed.), *Oxidative Stress and the Molecular Biology of Antioxidant Defenses*, Cold Spring Harbor Laboratory Press, Raleigh, USA, 1997.
3. J. M. McCord and I. Fridovich, *J. Biol. Chem.*, **244**, 6049 (1969).
4. J. A. Tainer, E. D. Getzoff, K. M. Beem, J. S. Richardson and D. C. Richardson, *J. Mol. Biol.,* **160**, 181 (1982).
5. T. J. Lyons, A. Nersissian, H. Huang, H. Yeom, C. R. Nishida, J. A. Graden, E. Butler Gralla and J. Seleverstone Valentine, *JBIC,* **5**,189 (2000).
6. C.-M. Liu, R.-G. Xiong, X.-Z. You and Y.-J. Liu, *Polyhedron,* **15**, 4565 (1996).
7. E. Bienvenue, S. Choua, M.-A. Lobo-Recio, C. Marzin, P. Pacheco, P. Seta and G. Tarrago, *J. Inorg. Biochem.*, **57**, 157 (1995).
8. **a)** J.-I. Ueda, T. Ozawa, M. Miyazaki and Y. Foujiwara, *Inorg. Chim. Acta,* **214**, 29 (1993) **b)** J.-I. Ueda, T. Ozawa, M. Miyazaki and Y. Fujiwara, *J. Inorg. Biochem.*, **55**, 123 (1994) **c)** R. N. Patel and K. B. Pandeya, *J. Inorg. Biochem.*, **72**, 109 (1998) **d)** M. Casolaro, M. Chelli, M. Ginanneschi, F. Laschi, L. Messori, M. Muniz-Miranda, A. M. Papini, T. Kowalik-Jankowska and H. Kozlowski, *J. Inrog. Biochem.,* **89**, 181 (2002).
9. A. Myari, G. Malandrinos, Y. Deligiannakis, J. C. Plakatouras, N. Hadjiliadis, Z. Nagy and I. Sovago, *J. Inorg. Biochem.*, **85**, 253 (2001).
10. K. Várnagy, J. Szabó, I. Sóvágó, G. Malandrinos, N. Hadjiliadis, D. Sanna and G. Micera, *J. Chem. Soc., Dalton Trans.*, 467 (2000).
11. M. T. Beck and I. Nagypál, *Chemistry of Complex Equilibria*, Ellis Horwood, Chichester, UK, 1990.
12. D. Leggett (Ed.), *Computational Methods for the Determination of Stability Constan*ts, Plenum, New York, 1991.
13. H. Kozlowski, W. Bal, M. Dyba and T. Kowalik-Jankowska, *Coord. Chem Rev.*, **184**, 319 (1999).
14. P. Gockel, M. Gelinsky, R. Vogler and H. Vahrenkamp, *Inorg. Chim. Acta,* **272**, 115 (1998).
15. I. Török, T. Gadja, B. Gyurcsik, G. K. Tóth and A. Péter, *J. Chem. Soc. Dalton Trans.*, 1205 (1998).
16. E. Farkas, I. Sóvágó and A. Gergely, *J. Chem. Soc. Dalton Trans.*, 1545 (1983).

17. D. L. Rabenstein and G. Blankey, *Inorg. Chem.*, 128 (1973).
18. D. L. Rabenstein, S. A. Daignault, A. A. Isab, A. P. Arnold and M. M. Shoukry, *J. Am. Chem. Soc.*, **107**, 6435 (1985).
19. S. A. Daignault, A. P. Arnold, A. A. Isab and D. L. Rabenstein, *Inorg. Chem.*, **24**, 3984 (1985).
20. P. G. Daniele, O. Zerbinati, R. Aruga and G. Ostacoli, *J. Chem. Soc. Dalton Trans.*, 1115 (1988).
21. C. E. Livera, L. D. Pettit, M. Bataille, B. Perly, H. Kozlowski and B. Radomska, *J. Chem. Soc. Dalton Trans.*, 661 (1987).
22. H. Aiba, A. Yokoyama and H. Tanaka, *Bull. Chem. Soc. Japan*, **47**, 136 (1974).
23. I. Sóvágó, E. Farkas and A. Gergely, *J. Chem. Soc. Dalton Trans.*, 2159 (1982).
24. J.-F. Galey, B. Decock-Le Reverend, A. Lebkiri, L. D. Pettit, S. I. Pyburn and H. Kozlowski, *J. Chem. Soc. Dalton Trans.*, 2281 (1991).
25. I. Sóvágó, D. Sanna, A. Dessi, K. Várnagy and G. Micera, *J. Inorg. Biochem.*, **63**, 99 (1996).

